# The Availability of Advanced Airway Equipment and Experience with Videolaryngoscopy in the UK: Two UK Surveys

**DOI:** 10.1155/2015/152014

**Published:** 2015-01-05

**Authors:** Rachel L. Gill, Audrey S. Y. Jeffrey, Alistair F. McNarry, Geoffrey H. C. Liew

**Affiliations:** ^1^Department of Anaesthesia, Western General Hospital, NHS Lothian, Crewe Road South, Edinburgh EH4 2XU, UK; ^2^Department of Anaesthesia, St. John's Hospital, NHS Lothian, Livingston EH54 6PP, UK; ^3^Department of Anaesthesia, Royal Infirmary of Edinburgh, Edinburgh EH16 4SA, UK; ^4^Department of Anaesthesia, Singapore General Hospital, Singapore 169608

## Abstract

Fibreoptic intubation, high frequency jet ventilation, and videolaryngoscopy form part of the Royal College of Anaesthetists compulsory higher airway training module. Curriculum delivery requires equipment availability and competent trainers. We sought to establish (1) availability of advanced airway equipment in UK hospitals (Survey I) and (2) if those interested in airway management (Difficult Airway Society (DAS) members) had access to videolaryngoscopes, their basic skill levels and teaching competence with these devices and if they believed that videolaryngoscopy was replacing conventional or fibreoptic laryngoscopy (Survey II). Data was obtained from 212 hospitals (73.1%) and 554 DAS members (27.6%). Most hospitals (202, 99%) owned a fiberscope, 119 (57.5%) had a videolaryngoscope, yet only 62 (29.5%) had high frequency jet ventilators. DAS members had variable access to videolaryngoscopes with Airtraq 319 (59.6%) and Glidescope 176 (32.9%) being the most common. More DAS members were happy to teach or use videolaryngoscopes in a difficult airway than those who had used them more than ten times. The majority rated Macintosh laryngoscopy as the most important airway skill. Members rated fibreoptic intubation and videolaryngoscopy skills equally. Our surveys demonstrate widespread availability of fibreoptic scopes, limited availability of videolaryngoscopes, and limited numbers of experienced videolaryngoscope tutors.

## 1. Introduction

The Royal College of Anaesthetists (RCoA) higher training module in airway management (compulsory for all UK trainees) was introduced in 2010. It is both detailed and specific in outlining the training required. Techniques of advanced fibreoptic intubation, high frequency jet ventilation, and videolaryngoscopy are all included [1].

We established in 2007 that final year trainees were not achieving their own definition of competence in awake fibreoptic intubation and Cook has highlighted the limited opportunities for training in the elective use of high frequency jet ventilation in laryngeal surgery [2, 3].

There are at least two components required to provide training with any piece of equipment. Firstly, the equipment must be available and secondly there must be sufficient trainers skilled with the device. Recent publications on videolaryngoscopy demonstrate its potential efficacy in the anticipated difficult airway situation and in the morbidly obese [4–7]. They have been shown to be easier for novices to use compared to the standard Macintosh laryngoscope and easier to use in airway teaching (due to shared trainer and trainee view) [8, 9]. Given this and their presence on the higher airway syllabus, these devices should be widely available.

As the syllabus is specific, we hypothesized that curriculum delivery would depend on (i) the availability of equipment and (ii) sufficient competent trainers.

The goals of our study were

- to determine whether departments had access to the equipment required to deliver the advanced airway techniques described,
- to determine if training in videolaryngoscopy, as required by the syllabus, would be deliverable,
- to determine the perceived status of videolaryngoscopy in the management of the difficult airway.

## 2. Materials and Methods

We conducted two surveys: survey I to establish equipment availability within hospitals in the UK and survey II (18 months later) to establish expertise among the members of the UK Difficult Airway Society (DAS). This organization, whilst self-selecting, represents those with a specific interest in airway management. As both surveys were surveys of equipment and staff practice (with no patient details or personal information), NHS ethical review was not required. Both surveys were piloted amongst a small group of anaesthetic colleagues across the UK; their pilot responses are not included in the analysis.

### 2.1. Survey I: Equipment Availability

We used the Directory of Operating Theatres and Departments of Surgery 2008 (Association for Perioperative Practice, Harrogate, UK) to provide a comprehensive list of UK NHS hospitals. We excluded isolated dental clinics and electroconvulsive therapy suites. All other NHS hospitals (both teaching and district general hospitals) were included as they can be involved in curriculum delivery and training. As private hospitals are not involved in the training of junior anaesthetists in the UK, we did not contact them. This methodology has been used by other surveys and audits [2, 3, 10].

We developed a standardised questionnaire, initially deployed in Scotland. We aimed to investigate the availability and number of four pieces of advanced airway equipment: (i) flexible fibreoptic laryngoscopes, (ii) videolaryngoscopes, (iii) low frequency jet ventilators (LFJV), and (iv) high frequency jet ventilators (HFJV). Starting in August 2010, all units in Scotland were telephoned and questions directed to the lead Operating Department Practitioner (ODP) or anaesthetist with an airway interest in their department. In October 2010, an identical postal questionnaire with postage-paid reply envelope was sent to all the college tutors in England, Wales, and Northern Ireland. The tutor was asked to complete or pass the questionnaire on to a colleague with an airway interest. Postal nonresponders were contacted by telephone after four weeks. Again, Senior ODPs or other available staff with appropriate knowledge of the equipment were sought to complete the questionnaire. We excluded hospitals that did not reply after three phone calls or where a valid telephone number for theatres was unobtainable. The same questions were asked by telephone as were on the postal questionnaire. A prize draw was offered to maximise the response rate. The questions and data are included in Supplementary Digital Content (see Supplementary Material available online at http://dx.doi.org/10.1155/2015/152014).

### 2.2. Survey II: Expert Experience with Videolaryngoscopy

We aimed to establish if those with a specific interest in airway management (as defined by their membership of the Difficult Airway Society (DAS)) had access to videolaryngoscopy, what experience they had of this skill, and whether they felt competent to teach it.

We distributed this survey to all members of DAS (members with a valid e-mail address, regardless of grade or country of residence) through the DAS Survey Coordinator (surveys@das.uk.com). An electronic questionnaire with 37 questions was created using Zoomerang Survey Software (http://www.zoomerang.com/) (Supplementary Digital Content shows the questions asked in the questionnaire and the responses given). We asked about the availability of videolaryngoscopes (all those commercially available in the UK at the time of the survey) and the respondents experience in their use and willingness to teach others to use them. The survey was modified once on the day of release to include a staff and associate specialist (SAS) category. Staff and Associate Specialist is the title given to non-consultant career grade doctors (not trainees) who can be involved in training delivery. Trainee anaesthetists complete a 7-year training programme identified as CT1, 2 in their first two years and ST3–7 in the final five years. The higher airway curriculum should be addressed in training years 5−7.

We also gave three statements regarding relative usefulness of advanced airway devices to be scored on a numerical scale of disagreement to agreement.

*Statement 1.* Learning how to use a supraglottic airway effectively is more important than learning how to use a Macintosh laryngoscope (completely disagree 0; completely agree 10).

*Statement 2.* Learning how to use a videolaryngoscope effectively is more important than learning how to use a Macintosh laryngoscope. 

*Statement 3.* Learning how to use a flexible fibrescope effectively in awake patients is more important than learning how to use a videolaryngoscope. The program created an 11-point scale (0–10).

The survey was open for four weeks and a single, centrally generated, reminder email was sent to all 2015 DAS members half way through the survey period.

### 2.3. Data Analysis

The data in Survey I was manually entered into a Microsoft Excel spreadsheet.

The data in Survey II was electronically downloaded to Microsoft Excel. The source IP addresses were provided by the survey and were inspected for duplication. Duplications were expected because of the potential for shared routers within a department. Responses from duplicated IP addresses were visually inspected to ensure there were no duplicated responses. Whilst this method cannot be 100% reliable, coupled with the absence of a return incentive and the length of the questionnaire, we believe that repeated responses are unlikely.

Analysis where appropriate was carried out using GraphPad Prism Version 6.03.

## 3. Results

### 3.1. Survey I

We contacted 290 hospitals in the United Kingdom which met the criteria. We collated responses from 212 units, a 73.1% response rate, with fully completed questionnaires from 204 units (70.3%). Of the 204 respondents, we found that 202 sites (99.0%) had at least one fibreoptic scope. The median fibreoptic scope: theatre ratio (median [range]) was 0.2 [0–3.0] across 512 theatre suites. Of the 210 respondents, 187 sites (89.0%) had their own difficult airway trolleys standardised across theatre suites.

Regarding the availability of videolaryngoscopes, 119 sites of 207 (57.5%) reported having videolaryngoscopes (Table 2). Most sites (104 of 119, 87.4%) owned the available devices; the rest were on loan from the manufacturers and six hospitals which owned videolaryngoscopes were trialing additional devices. Not all hospitals had access to a videolaryngoscope for routine use (106 of 119 sites (89.0%)).

Low frequency jet ventilators were available on the difficult airway trolley on 183 sites (87.1%). Seventy-two of those sites (39.3%) had additional low frequency jet ventilators for elective use; in 44 (61.1%, *n* = 72) of these sites, it was their only available technique for elective use. High frequency jet ventilators were reported as available in 62 sites (29.5%). One hundred and fifty-one units (71.9%) had ENT, maxillofacial or head and neck services on-site.

### 3.2. Survey II

Five hundred and fifty-four responses were received, a response rate of 27.5%, of which 469 (84.7%) were complete. DAS is open to membership from all countries; however the majority of respondents were from the UK (535, 97.4%). All deaneries and all grades of anaesthetist were represented in the survey and the demographic details are summarized in Table 1. Eighty-two respondents (14.8%, *n* = 553) had led the procurement process for videolaryngoscopy and a further 164 (29.7%) had been involved in it. Two hundred and thirty-eight (43.0%, *n* = 553) had an operating session where “difficult airways” are encountered regularly. Not all respondents completed all fields, explaining the variable denominator; however all responses are included in the analysis. Five hundred and thirty-five respondents confirmed they were UK based members of DAS and the analysis of videolaryngoscope availability is based on their responses.

The Airtraq (319, 59.6%), Glidescope (176, 32.9%), and CMAC (110, 20.5%) were the videolaryngoscopes that most respondents (*n* = 535) had access to. The number of individuals who were happy to use these devices in potentially difficult airways and to teach others to use these devices was consistently higher than the number who had used the device more than ten times. The sole exception to this was the Kingvision which had recently been marketed, for which there were a small number of responses. These results are in Table 2. Given this, we reanalysed the data to see how many people with ready access to a device, and who had used it more than ten times, were happy to teach others. These results are shown in Table 3.

**Figure 1:**
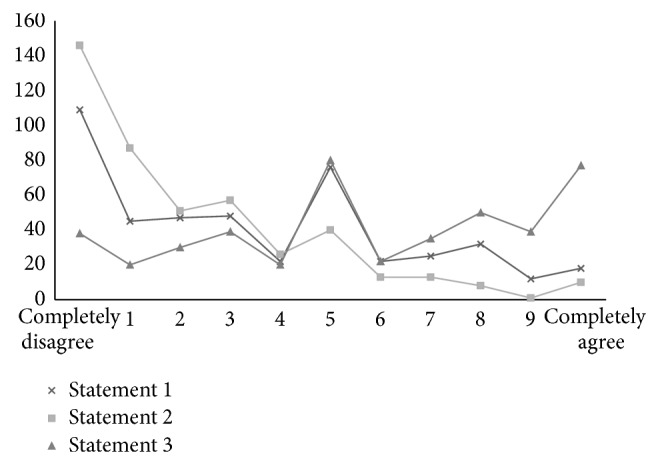
Survey II respondents rating of three statements concerning Macintosh, videolaryngoscopy and fibreoptic laryngoscopy. Learning to use supraglottic devices is more important than learning intubation with Macintosh laryngoscope: Statement 1. Learning to use videolaryngoscope is more important than learning intubation with Macintosh laryngoscope: Statement 2. Learning to use fibrescope in awake patients is more important than learning to use videolaryngoscope: Statement 3.

The final three questions in the 2nd questionnaire showed a spectrum of opinions from all 554 respondents (the program would not allow separation of non-UK data in this section, *n* = 22). Respondents rated each statement from 0 to 10 where 0 was complete disagreement and 10 was complete agreement (Figure 1). Respondents felt learning to use supraglottic devices was not more important than learning intubation with Macintosh laryngoscope (average 3.51). They also believed that learning to use a videolaryngoscope was not more important than learning intubation with a Macintosh laryngoscope (average 2.23), suggesting greater disagreement with Statement 2 than Statement 1. On the final question, respondents were approximately equally divided (average 5.72) on whether they disagreed (0) or agreed (10) with the statement that “learning to use a flexible fibrescope in awake patients is more important than learning how to use a videolaryngoscope.”

The complete results of questionnaire 2 are included as Supplementary Digital Content.

## 4. Discussion

We conducted these surveys to establish the availability of advanced airway devices as listed in the Royal College of Anaesthetists (RCoA) 2010 syllabus mandatory higher airway training module [1]. This specifically identifies skills in fibreoptic intubation, high frequency jet ventilation and videolaryngoscopy and we focused our questions accordingly. All of these techniques require not just operator skill but also equipment availability and competent trainers.

Previous studies have already demonstrated the lack of availability of HFJV and poor training in fibreoptic laryngoscopy [2, 3]. The 4th National Audit Project of the RCoA and DAS (NAP4) specifically mentions the theoretical though unproven benefit of videolaryngoscopes in turning blind intubations into visualised intubations [11]. There is now evidence that videolaryngoscopes can function more effectively than a conventional Macintosh laryngoscope; however the availability of videolaryngoscopes across the UK has yet to be investigated [4–7].

We found that videolaryngoscopes were not available in all hospitals in 2010 or to all individuals responding in 2012. This is an impediment to training. Equally important was our finding that, in hospitals where certain devices were readily available, the number of anaesthetists who had used the videolaryngoscope more than 10 times and were willing to teach the use of the device constituted only a small proportion of the total number of respondents.

Experience of videolaryngoscopy did not dictate perceived teaching ability—this is concerning. More DAS members were happy to teach or use videolaryngoscopes in a difficult airway than those who had used them more than ten times. Previous studies on novices and experienced anaesthetists have suggested that, across a range of videolaryngoscope devices, around 20 uses may be required to become competent with a device [12]. Although these figures are less than those suggested by Greaves (80% competence with 30 cases, with continued improvement over 100 cases), the video-imaging technology of these new devices offers a shared view between trainer and trainee [9, 13]. This can facilitate teaching of airway anatomy, critical appraisal of technique, and feedback. This can lead to more rapid skill acquisition than is achievable with traditional training with direct laryngoscopy [9, 14]; however we were concerned by the willingness of several respondents to teach a device they themselves had not used more than ten times. This is supported by the data in Table 3, where, when respondents had used a device more than ten times, they were not necessarily confident to teach it. We have already identified that simply having a device does not necessarily lead to adequate training. We found that 99.0% of theatre suites had a fibrescope but we know that in 2007 inadequate training existed [2]. Recent studies of videolaryngoscopes demonstrate an improved Cormack and Lehane view but did not identify the best device [5–7, 15–19]. This lack of “best videolaryngoscope” may explain diverse availability of devices across the UK. The widespread availability of the Airtraq (released to the UK in 2006) may be because of its early release date or because of its low individual unit acquirement cost. We were concerned to discover that there were hospitals with videolaryngoscopes which theatre staff could not identify by name. Several hospitals both in 2010 and in 2012 had videolaryngoscopes which were not readily available for use and were kept only for specific situations. If there is already an educational issue around the availability of these devices, then this limited utilisation will simply compound this. Varied availability of videolaryngoscopes (nine types in 2012 survey) will expose trainees to different devices; however it may mean that minimal expertise is gained with any particular device.

We recognise that one method of assessment of the use of videolaryngoscopes is through simulation [20]. There are obvious benefits of learning through simulation and whilst McFetrich has suggested that simulation can be useful in procedures that are performed rarely, we believe that videolaryngoscopes do not fall into this category and should be used regularly. Cook and Alexander's national survey conducted in 2006 reported a HFJV availability of 7.1% [3]. Our survey in 2010 reported an availability of 29.3%. This may represent widespread take-up of the technology but may also be misreporting of similar anaesthetic equipment as that shown by those unable to identify the videolaryngoscope in their department. We are also concerned that some units only had a low frequency jet ventilator available for elective use—this means that training in HFJV techniques will be limited. It also contradicts Biro's advice who suggests that low cost manual jet ventilators such as the manujet are not recommended as “standard equipment in upper airway surgery” [21].

Respondents may have felt uncomfortable providing information that portrayed their institution in an unfavourable light—this may have contributed to the number of units reporting to have HFJV. In an airway emergency, all members of the theatre team have an important contribution to make. If staff cannot readily identify the equipment or do not know how it operates, there could be significant implications for clinical outcome. Clearly, all staff should receive appropriate airway training as highlighted in the NAP4 report for team training in airway management.

We also invited opinion on the relative importance of different airway skills. Interestingly, the DAS respondents rated learning direct laryngoscopy effectively as more important than learning to use supraglottic devices. Cook's 2006 editorial highlighted that novice trainees intubated on average one patient per working day and performed one rapid sequence induction (RSI) per working week [22]. Studies showed that novice trainees needed to have performed 47−57 intubations to have a 90% chance of successful intubation, whereas the learning curve of a supraglottic device was cited to be between 75 and 750 insertions [23–26]. The role of training in supraglottic airway device insertion is important because successful insertion of an appropriate supraglottic airway is the DAS strategy for failed intubation and NAP4 reported incorrect use of a supraglottic airway [27].

Given our findings on the availability of videolaryngoscopes, we were unsurprised to find that respondents disagreed most strongly with Statement 2 (learning to use a videolaryngoscope effectively is more important than learning intubation with a Macintosh laryngoscope).

However, in light of the survey's principal findings (widespread availability of fibreoptic scopes, limited availability of videolaryngoscopes, and limited numbers of experienced videolaryngoscope tutors), we were surprised by the responses to Statement 3. DAS members were essentially equally divided when it came to choosing between training in an awake FOI technique and training in videolaryngoscope use. Whilst NAP4 reports that awake fibreoptic intubation can fail, we are concerned by the willingness of respondents to equate videolaryngoscopy with this proven technique of difficult airway management. DAS members had frequently not used their available videolaryngoscopes more than ten times. Of those that had done so, fewer were confident to teach it (CMAC excepted).

Surveys have limitations. We recognise that all surveys are snapshots of opinion and are dependent not just on the response rate but on the accuracy of information provided by the respondents. Even the choice of language can influence the responses given although we would have hoped that any specific issues would have been addressed by our pilot audience. The response rate in Survey I is high although there are more respondents in Survey II with a lower response rate. It could be argued that this prevents us from drawing meaningful conclusions from Survey II data; however we contest that it is more likely to be active and enthusiastic members of DAS who made the effort to complete a 37-question online survey. Similarly, we cannot reflect the views of those who did not participate in the survey. However, the respondents views highlight some of the challenges around effective education in advanced airway devices.

We accept that our work is exploratory and serves to identify the baseline situation when the surveys were conducted. Given the apparently disparate results, further investigation will be required to determine which tools should be available, taught, and mastered in the operating theatre environments and whether specific individual devices could or should be mandated by a national standard such as the curriculum.

As we have found an apparent lack of trainers who have used videolaryngoscopes frequently, we are keen to investigate how an institution learns a new device and how the success of that learning may be measured. Whilst the ADEPT process [28] may determine which device we should use, it is not yet clear how best individuals should learn a new device or how to retain such skills with both mannequin and clinical situations having been used [29, 30].

## 5. Conclusion

Our surveys demonstrate widespread availability of fibreoptic scopes and limited access to high frequency jet ventilators and limited availability of videolaryngoscopes with limited numbers of experienced videolaryngoscopy tutors. We are concerned by the willingness of airway enthusiasts to favour newer less available techniques with which they have less experience.

Advanced airway techniques will continue to evolve. The challenge that exists is to effectively educate the next generation of anaesthetists in their safe and appropriate use. This must include competent trainers who have sufficient access to the devices to maintain their skills and educate others.

## Supplementary Material

The Supplementary Material shows the questions asked and answers provided in Questionnaires 1 and 2. In Questionnaire 1 the responses are divided into responses from Scotland and England Wales and Northern Ireland responses as this is how the questionnaire was distributed. The responses displayed in Questionnaire 2 are as created by Zoomerang Survey Software (now Survey Monkey).

## Figures and Tables

**Table:** (a) Respondents of Survey II by country, *n* = 549

Country	UK	Republic of Ireland	Other EU	America and Canada	Australia and New Zealand	Other
Respondents (%)	535 (97.4)	1 (0.2)	1 (0.2)	3 (0.5)	6 (1.1)	3 (0.5)

**Table:** (b) Grade of respondent, *n* = 547

Grade of respondent	CT1, 2	ST3, 4	ST5, 6, 7	Consultant or SAS years' experience				
				0–<5	5–<10	10–<15	15–<20	>20
Response (%)	9 (1.6)	24 (4.4)	110 (20.1)	156 (28.5)	80 (14.6)	84 (15.4)	43 (7.9)	41 (7.5)

**Table 2:** Videolaryngoscopy availability data from Surveys I and II and UK device release date.

List of devices	Date available to UK	Number of hospitals with device available in 2010	Respondents with device available for use in 2012 Number (percentage)	Respondents who have used device more than 10 times	Respondents who are happy to use the device in a potentially difficult airway	Respondents who are confident to teach use of the device	Respondents who are not happy to use this device
Airtraq	2006	40 (19.3)	319 (59.6)	147 (27.5)	191 (35.7)	188 (35.1)	110 (20.6)
AP Advance	2011		61 (11.4)	24 (4.5)	50 (9.3)	44 (8.2)	297 (55.5)
Bonfils^*^	2005	3 (1.4)	102 (19.1)^*^	34 (6.4)	50 (9.3)	39 (7.3)	282 (52.7)
C-MAC	2008	5 (2.4)	110 (20.5)	71 (13.3)	101 (18.9)	105 (19.6)	214 (40.0)
Glidescope	2009	24 (11.6)	176 (32.9)	143 (26.7)	156 (29.2)	146 (27.3)	129 (24.1)
King Vision	2011		4 (0.7)	5 (0.9)	4 (0.7)	6 (1.1)	348 (65.0)
McGrath Series 5	2006	20 (9.7)	67 (12.5)	47 (8.8)	51 (9.5)	56 (10.5%)	247 (46.2)
McGrath MAC	2010		47 (8.8)	36 (6.7)	41 (7.7)	46 (8.6)	256 (47.9)
Pentax AWS	2006	15 (7.2)	46 (8.3)	35 (6.5)	52 (9.7)	46 (8.6)	263 (49.2)
Shikani^†^	1999	2 (1.0)	7 (1.3)				
Not specified		42 (20.3)					

Results are number of respondents (%). Denominators 207 respondents Survey I (2010) [some hospitals had more than 1 device], 535 respondents Survey II (2012).

^*^In Survey II, all optical stylets were included in a “Bonfils or other optical stylet” category. Where respondents specifically identified the Shikani, this was listed additionally ^†^to allow comparison with the 2010 data where Shikani was identified by 2 respondents. The Shikani availability date is when it was available in the USA.

**Table 3:** DAS respondents who had ready access to a device, had used it more than ten times, and were confident to teach its use (2012 survey).

Device	Readily available	Has used >10 times	Confident to teach
Airtraq	231	98 (42.4)	80 (34.6)
AP advance	32	11 (34.4)	7 (21.9)
Bonfils	58	14 (24.1)	9 (15.5)
CMAC	80	9 (11.3)	9 (11.3)
Glidescope	143	41 (28.7)	32 (22.4)
Kingvision	0	0	0
Series 5	39	25 (64.1)	20 (51.3)
MAC	38	22 (57.9)	17 (44.7)
Pentax AWS	32	21 (65.6)	17 (53.1)

*n*: number of respondents (%); percentages in the 2nd and 3rd column are of those who had the device readily available.
