# Supplementary material for: The Availability of Advanced Airway Equipment and Experience with Videolaryngoscopy in the UK: Two UK Surveys
Source: Anesthesiol Res Pract. 2015 Jan 5;2015:152014. doi: 10.1155/2015/152014 (PMC4299561; doi:10.1155/2015/152014)
Supplement: Supplementary file 1 — The Supplementary Material shows the questions asked and answers provided in Questionnaires 1 and 2. In Questionnaire 1 the responses are divided into responses from Scotland and England Wales and Northern Ireland responses as this is how the questionnaire was distributed. The responses displayed in Questionnaire 2 are as created by Zoomerang Survey Software (now Survey Monkey). [file 152014.f1.docx]

# SDC –questionnaire 1

|  | **Yes Response Percent** | **Yes Response Count** | **Scotland** | **England Wales and Northern Ireland** |
| --- | --- | --- | --- | --- |
| Survey sent |  | 290 | 43 | 247 |
| Survey response | 73.1 | 212 | 43 | 169 |
| Response complete | 70.3 | 204 | 43 | 161 |
| Response partially complete | 2.8 | 8 | 0 | 8 |
| **Q1** Do you have fibreoptic scopes | 99.0 | 202 | 42 | 160 |
| **Q2** Do you have any videolaryngoscopes? | 57.5 | 119 | 23 | 96 |
| **Q3** Owned? | 87.4 | 104 | 15 | 89 |
| **Q4** On trial? | 12 | 20 | 8 | 12 |
| Owned & on trial |  |  | 0 | 6 |
| **Q5** Are they for routine use and emergencies | 85.7 | 102 | 20 | 82 |
| Routine use only | 3.4 | 4 | 0 | 4 |
| Emergencies only | 10.1 | 12+1locked up | 2+1locked up | 10 |
| **Q6** Are your difficult airway trolleys standardised across the theatre suites? | 89.0 | 187 | 43 | 145 |
| **Q7** Do you have a high frequency jet ventilator? | 29.5 | 62 | 4 | 58 |
| **Q8** Does the difficult airway trolley have LFJV eg manujet? | 87.1 | 183 | 37 | 146 |
| **Q9** Do you have separate low frequency jet ventilation eg. manujet for elective situations? | 34.3 | 72 | 14 | 58 |
| **Q10** Do you have Head & Neck/ MaxFax/ ENT services on this site? | 71.9 | 151 | 26 | 125 |

| **Q1 What Grade of anaesthetist are you** | | |
| --- | --- | --- |
| **Answer Options** | **Response Percent** | **Response Count** |
| CT1,2 | 1.6% | 9 |
| ST3,4 | 4.4% | 24 |
| ST5,6,7 | 20.1% | 110 |
| Consultant or SAS 0- <5 years experience | 28.5% | 156 |
| Consultant or SAS 5- <10 years experience | 14.6% | 80 |
| Consultant or SAS 10- <15 years experience | 15.4% | 84 |
| Consultant or SAS 15- <20 years experience | 7.9% | 43 |
| Consultant or SAS >20 years experience | 7.5% | 41 |
| ***answered question*** | | **547** |
| ***skipped question*** | | **7** |

# SDC –questionnaire 2

| **Q2 Where do you work** | | |
| --- | --- | --- |
| **Answer Options** | **Response Percent** | **Response Count** |
| UK | 97.4% | 535 |
| Republic of Ireland | 0.2% | 1 |
| Other EU | 0.2% | 1 |
| America and Canada | 0.5% | 3 |
| Australia and New Zealand | 1.1% | 6 |
| Other | 0.5% | 3 |
| ***answered question*** | | **549** |
| ***skipped question*** | | **5** |

| **Q3 If you work in the UK would you please specify your deanery** | | |
| --- | --- | --- |
| **Answer Options** | **Response Percent** | **Response Count** |
| Unsure | 0.8% | 4 |
| Defence | 0.6% | 3 |
| East Midlands | 6.0% | 32 |
| East of England | 7.0% | 37 |
| Kent, Surrey, Sussex | 6.4% | 34 |
| London | 18.7% | 99 |
| Mersey | 1.1% | 6 |
| North Western | 7.6% | 40 |
| Northern | 4.0% | 21 |
| NIMDTA (Northern Ireland) | 1.5% | 8 |
| Oxford | 3.4% | 18 |
| Scotland East | 3.0% | 16 |
| Scotland North | 1.3% | 7 |
| Scotland South East | 2.1% | 11 |
| Scotland West | 4.5% | 24 |
| Severn | 3.8% | 20 |
| South West Peninsula | 3.0% | 16 |
| Wales | 6.0% | 32 |
| Wessex | 2.6% | 14 |
| West Midlands | 8.9% | 47 |
| Yorkshire and the Humber | 7.6% | 40 |
| ***answered question*** | | **529** |
| ***skipped question*** | | **25** |

| **Q4 Have you in the last five years been involved in the selection of videolaryngoscopes to stock in your hospital** | | |
| --- | --- | --- |
| **Answer Options** | **Response Percent** | **Response Count** |
| Yes led process | 14.8% | 82 |
| Yes, gave opinions | 29.7% | 164 |
| No | 55.5% | 307 |
| ***answered question*** | | **553** |
| ***skipped question*** | | **1** |

| **Q5 Do you do a list where 'difficult airways' are encountered regularly** | | |
| --- | --- | --- |
| **Answer Options** | **Response Percent** | **Response Count** |
| Yes | 43.0% | 238 |
| No | 57.0% | 315 |
| ***answered question*** | | **553** |
| ***skipped question*** | | **1** |

| **Q6 About the Airtraq availability** | | |
| --- | --- | --- |
| **Answer Options** | **Response Percent** | **Response Count** |
| These are readily available for use in our hospital | 48.5% | 237 |
| These are available for specific cases on a limited bases | 18.8% | 92 |
| These are unavailable in our hospital and I am happy with that | 21.5% | 105 |
| These are unavailable in our hospital but I would like them to be | 11.2% | 55 |
| ***answered question*** | | **489** |
| ***skipped question*** | | **65** |

| **Q7 About your experience with the airtraq** | | |
| --- | --- | --- |
| **Answer Options** | **Response Percent** | **Response Count** |
| I have never used the device | 16.1% | 86 |
| I have used it between 1 and 10 times | 55.7% | 297 |
| I have used it more than 10 times | 28.1% | 150 |
| ***answered question*** | | **533** |
| ***skipped question*** | | **21** |

| **Q8 About the Airtraq, teaching and training** | | |
| --- | --- | --- |
| **Answer Options** | **Response Percent** | **Response Count** |
| I am happy to use this device | 58.7% | 310 |
| I am happy to use this device in a potentially difficult airway | 36.7% | 194 |
| I feel confident to teach others to use this device | 36.6% | 193 |
| I am not happy to use this device | 21.2% | 112 |
| ***answered question*** | | **528** |
| ***skipped question*** | | **26** |

| **Q9 About the AP Advance availability** | | |
| --- | --- | --- |
| **Answer Options** | **Response Percent** | **Response Count** |
| It is readily available for use in our hospital | 7.3% | 32 |
| It is available for specific cases on a limited bases | 6.8% | 30 |
| It is unavailable in our hospital and I am happy with that | 60.3% | 264 |
| It is unavailable in our hospital but I would like it to be | 25.6% | 112 |
| ***answered question*** | | **438** |
| ***skipped question*** | | **116** |

| **Q10 About your experience with the AP Advance** | | |
| --- | --- | --- |
| **Answer Options** | **Response Percent** | **Response Count** |
| I have never used the device | 75.9% | 382 |
| I have used it between 1 and 10 times | 19.3% | 97 |
| I have used it more than 10 times | 4.8% | 24 |
| ***answered question*** | | **503** |
| ***skipped question*** | | **51** |

| **Q11 About the AP Advance, teaching and training** | | |
| --- | --- | --- |
| **Answer Options** | **Response Percent** | **Response Count** |
| I am happy to use this device | 23.3% | 104 |
| I am happy to use this device in a potentially difficult airway | 11.4% | 51 |
| I feel confident to teach others to use this device | 10.1% | 45 |
| I am not happy to use this device | 69.4% | 310 |
| ***answered question*** | | **447** |
| ***skipped question*** | | **107** |

| **Q12 About the Bonfils availability** | | |
| --- | --- | --- |
| **Answer Options** | **Response Percent** | **Response Count** |
| It is readily available for use in our hospital | 12.7% | 59 |
| It is available for specific cases on a limited bases | 9.9% | 46 |
| It is unavailable in our hospital and I am happy with that | 53.5% | 249 |
| It is unavailable in our hospital but I would like it to be | 23.9% | 111 |
| ***answered question*** | | **465** |
| ***skipped question*** | | **89** |

| **Q13 If you access to an alternative optical stylet (eg Levitan or Shikani) please enter it here and answer the questions below in relation to the device you do have** | |
| --- | --- |
| **Answer Options** | **Response Count** |
|  | 19 |
| ***answered question*** | **19** |
| ***skipped question*** | **535** |

| **Q14 About your experience with the bonfils** | | |
| --- | --- | --- |
| **Answer Options** | **Response Percent** | **Response Count** |
| I have never used the device | 61.0% | 297 |
| I have used it between 1 and 10 times | 31.8% | 155 |
| I have used it more than 10 times | 7.2% | 35 |
| ***answered question*** | | **487** |
| ***skipped question*** | | **67** |

| **Q15 About the bonfils, teaching and training** | | |
| --- | --- | --- |
| **Answer Options** | **Response Percent** | **Response Count** |
| I am happy to use this device | 27.4% | 122 |
| I am happy to use this device in a potentially difficult airway | 11.4% | 51 |
| I feel confident to teach others to use this device | 9.4% | 42 |
| I am not happy to use this device | 65.5% | 292 |
| ***answered question*** | | **446** |
| ***skipped question*** | | **108** |

| **Q16 About the CMAC availability** | | |
| --- | --- | --- |
| **Answer Options** | **Response Percent** | **Response Count** |
| It is readily available for use in our hospital | 19.2% | 86 |
| It is available for specific cases on a limited bases | 7.4% | 33 |
| It is unavailable in our hospital and I am happy with that | 44.5% | 199 |
| It is unavailable in our hospital but I would like it to be | 28.9% | 129 |
| ***answered question*** | | **447** |
| ***skipped question*** | | **107** |

| **Q17 About your experience with the CMAC** | | |
| --- | --- | --- |
| **Answer Options** | **Response Percent** | **Response Count** |
| I have never used the device | 55.5% | 264 |
| I have used it between 1 and 10 times | 29.2% | 139 |
| I have used it more than 10 times | 15.3% | 73 |
| ***answered question*** | | **476** |
| ***skipped question*** | | **78** |

| **Q18 About the CMAC, teaching and training** | | |
| --- | --- | --- |
| **Answer Options** | **Response Percent** | **Response Count** |
| I am happy to use this device | 37.5% | 165 |
| I am happy to use this device in a potentially difficult airway | 24.5% | 108 |
| I feel confident to teach others to use this device | 25.0% | 110 |
| I am not happy to use this device | 49.5% | 218 |
| ***answered question*** | | **440** |
| ***skipped question*** | | **114** |

| **Q19 About the Glidescope availability** | | |
| --- | --- | --- |
| **Answer Options** | **Response Percent** | **Response Count** |
| It is readily available for use in our hospital | 33.8% | 153 |
| It is available for specific cases on a limited bases | 7.3% | 33 |
| It is unavailable in our hospital and I am happy with that | 35.4% | 160 |
| It is unavailable in our hospital but I would like it to be | 23.5% | 106 |
| ***answered question*** | | **452** |
| ***skipped question*** | | **102** |

| **Q20 About your experience with the Glidescope** | | |
| --- | --- | --- |
| **Answer Options** | **Response Percent** | **Response Count** |
| I have never used the device | 26.6% | 126 |
| I have used it between 1 and 10 times | 41.4% | 196 |
| I have used it more than 10 times | 32.1% | 152 |
| ***answered question*** | | **474** |
| ***skipped question*** | | **80** |

| **Q21 About the Glidescope teaching and training** | | |
| --- | --- | --- |
| **Answer Options** | **Response Percent** | **Response Count** |
| I am happy to use this device | 52.3% | 237 |
| I am happy to use this device in a potentially difficult airway | 36.0% | 163 |
| I feel confident to teach others to use this device | 33.8% | 153 |
| I am not happy to use this device | 28.7% | 130 |
| ***answered question*** | | **453** |
| ***skipped question*** | | **101** |

| **Q22 About the Kingvision availability** | | |
| --- | --- | --- |
| **Answer Options** | **Response Percent** | **Response Count** |
| It is readily available for use in our hospital | 0.0% | 0 |
| It is available for specific cases on a limited bases | 0.9% | 4 |
| It is unavailable in our hospital and I am happy with that | 78.7% | 343 |
| It is unavailable in our hospital but I would like it to be | 20.4% | 89 |
| ***answered question*** | | **436** |
| ***skipped question*** | | **118** |

| **Q23About your experience with the Kingvision** | | |
| --- | --- | --- |
| **Answer Options** | **Response Percent** | **Response Count** |
| I have never used the device | 97.0% | 450 |
| I have used it between 1 and 10 times | 1.9% | 9 |
| I have used it more than 10 times | 1.1% | 5 |
| ***answered question*** | | **464** |
| ***skipped question*** | | **90** |

| **Q24 About the Kingvision teaching and training** | | |
| --- | --- | --- |
| **Answer Options** | **Response Percent** | **Response Count** |
| I am happy to use this device | 6.4% | 25 |
| I am happy to use this device in a potentially difficult airway | 1.0% | 4 |
| I feel confident to teach others to use this device | 1.5% | 6 |
| I am not happy to use this device | 93.0% | 361 |
| ***answered question*** | | **388** |
| ***skipped question*** | | **166** |

| **Q25 About the McGrath Series 5 availability (McGrath MAC next Question)** | | |
| --- | --- | --- |
| **Answer Options** | **Response Percent** | **Response Count** |
| It is readily available for use in our hospital | 9.1% | 39 |
| It is available for specific cases on a limited bases | 7.0% | 30 |
| It is unavailable in our hospital and I am happy with that | 58.7% | 252 |
| It is unavailable in our hospital but I would like it to be | 25.2% | 108 |
| ***answered question*** | | **429** |
| ***skipped question*** | | **125** |

| **Q26 About your experience with the McGrath Series 5 (McGrath MAC next question)** | | |
| --- | --- | --- |
| **Answer Options** | **Response Percent** | **Response Count** |
| I have never used the device | 69.6% | 316 |
| I have used it between 1 and 10 times | 19.8% | 90 |
| I have used it more than 10 times | 10.6% | 48 |
| ***answered question*** | | **454** |
| ***skipped question*** | | **100** |

| **Q27 About the McGrath Series 5 teaching and training (McGrath MAC Next question)** | | |
| --- | --- | --- |
| **Answer Options** | **Response Percent** | **Response Count** |
| I am happy to use this device | 27.8% | 111 |
| I am happy to use this device in a potentially difficult airway | 12.8% | 51 |
| I feel confident to teach others to use this device | 14.5% | 58 |
| I am not happy to use this device | 64.2% | 256 |
| ***answered question*** | | **399** |
| ***skipped question*** | | **155** |

| **Q28 About the McGrath MAC availability (Not the series 5)** | | |
| --- | --- | --- |
| **Answer Options** | **Response Percent** | **Response Count** |
| It is readily available for use in our hospital | 9.1% | 38 |
| It is available for specific cases on a limited bases | 2.4% | 10 |
| It is unavailable in our hospital and I am happy with that | 64.4% | 269 |
| It is unavailable in our hospital but I would like it to be | 24.2% | 101 |
| ***answered question*** | | **418** |
| ***skipped question*** | | **136** |

| **Q29 About your experience with the McGrath MAC (Not the series 5)** | | |
| --- | --- | --- |
| **Answer Options** | **Response Percent** | **Response Count** |
| I have never used the device | 77.6% | 349 |
| I have used it between 1 and 10 times | 14.4% | 65 |
| I have used it more than 10 times | 8.0% | 36 |
| ***answered question*** | | **450** |
| ***skipped question*** | | **104** |

| **Q30 About the McGrath MAC teaching and training (not the series 5)** | | |
| --- | --- | --- |
| **Answer Options** | **Response Percent** | **Response Count** |
| I am happy to use this device | 24.5% | 95 |
| I am happy to use this device in a potentially difficult airway | 10.6% | 41 |
| I feel confident to teach others to use this device | 12.1% | 47 |
| I am not happy to use this device | 68.0% | 263 |
| ***answered question*** | | **387** |
| ***skipped question*** | | **167** |

| **Q31 About the Pentax AWS availability** | | |
| --- | --- | --- |
| **Answer Options** | **Response Percent** | **Response Count** |
| It is readily available for use in our hospital | 7.6% | 32 |
| It is available for specific cases on a limited bases | 3.3% | 14 |
| It is unavailable in our hospital and I am happy with that | 66.7% | 280 |
| It is unavailable in our hospital but I would like it to be | 22.4% | 94 |
| ***answered question*** | | **420** |
| ***skipped question*** | | **134** |

| **Q32 About your experience with the Pentax AWS** | | |
| --- | --- | --- |
| **Answer Options** | **Response Percent** | **Response Count** |
| I have never used the device | 75.4% | 340 |
| I have used it between 1 and 10 times | 16.6% | 75 |
| I have used it more than 10 times | 8.0% | 36 |
| ***answered question*** | | **451** |
| ***skipped question*** | | **103** |

| **Q33 About the Pentax AWS, teaching and training** | | |
| --- | --- | --- |
| **Answer Options** | **Response Percent** | **Response Count** |
| I am happy to use this device | 25.1% | 98 |
| I am happy to use this device in a potentially difficult airway | 13.8% | 54 |
| I feel confident to teach others to use this device | 11.8% | 46 |
| I am not happy to use this device | 69.2% | 270 |
| ***answered question*** | | **390** |
| ***skipped question*** | | **164** |

| **Q34 Please rate the following three statements: Learning how to use a supraglottic airway effectively is more important than learning how to intubate with a Macintosh Laryngoscope** | | | | | | | | | | | | | |
| --- | --- | --- | --- | --- | --- | --- | --- | --- | --- | --- | --- | --- | --- |
| **Answer Options** | **Completely Disagree  with statement "learning supraglottic airway more important"** |  |  |  |  |  |  |  |  |  | **Completely Agree with statement "learning supraglottic airway more important"** | **Rating Average** | **Response Count** |
|  | 109 | 45 | 47 | 48 | 22 | 76 | 22 | 25 | 32 | 12 | 18 | 3.51 | 456 |
| ***answered question*** | | | | | | | | | | | | | **456** |
| ***skipped question*** | | | | | | | | | | | | | **98** |

| **Q35 Learning how to use a videolaryngoscope effectively is more important than learning how to intubate with a Macintosh Laryngoscope** | | | | | | | | | | | | | |
| --- | --- | --- | --- | --- | --- | --- | --- | --- | --- | --- | --- | --- | --- |
| **Answer Options** | **Completely Disagree with statement  "learning how to use a videolaryngoscope is more important"** |  |  |  |  |  |  |  |  |  | **Completely Agree with statement "learning how to use a videolaryngoscope more important"** | **Rating Average** | **Response Count** |
|  | 146 | 87 | 51 | 57 | 26 | 40 | 13 | 13 | 8 | 1 | 10 | 2.23 | 452 |
| ***answered question*** | | | | | | | | | | | | | **452** |
| ***skipped question*** | | | | | | | | | | | | | **102** |

| **Q36 Learning how to use a flexible fibrescope effectively in awake patients is more important than learning how to intubate with a Videolaryngoscope** | | | | | | | | | | | | | |
| --- | --- | --- | --- | --- | --- | --- | --- | --- | --- | --- | --- | --- | --- |
| **Answer Options** | **Completely Disagree with statement"learning awake fibreoptic intubation is moreimportant than learning videolaryngoscopy"** |  |  |  |  |  |  |  |  |  | **Completely Agree with statement  "learning awake fibreoptic intubation is moreimportant than learning videolaryngoscopy"** | **Rating Average** | **Response Count** |
|  | 38 | 20 | 30 | 39 | 20 | 80 | 22 | 35 | 50 | 39 | 77 | 5.72 | 450 |
| ***answered question*** | | | | | | | | | | | | | **450** |
| ***skipped question*** | | | | | | | | | | | | | **104** |

| **Q37 ANYTHING you wish to add (Free text response)?** | |
| --- | --- |
| **Answer Options** | **Response Count** |
|  | 127 |
| ***answered question*** | **127** |
| ***skipped question*** | **427** |
